# Space Charges at SrTiO_3_|Mixed Ionic and Electronic Conducting Oxide Heterojunctions and Their Relation to Defect Chemistry

**DOI:** 10.1021/acsami.4c21843

**Published:** 2025-03-05

**Authors:** Claudia Steinbach, Alexander Schmid, Matthäus Siebenhofer, Andreas Nenning, Christoph Rameshan, Markus Kubicek, Juergen Fleig

**Affiliations:** † Institute of Chemical Technologies and Analytics, TU Wien, Vienna1060, Austria; ‡ Department of Nuclear Engineering, Massachusetts Institute of Technology, Cambridge, Massachusetts 02139, United States; ¶ Department General, Analytical and Physical Chemistry, Montanuniversität Leoben, Leoben 8700, Austria

**Keywords:** space charge, strontium titanate, mixed ionic electronic conductor, defect chemistry, electrochemical potential, reducibility

## Abstract

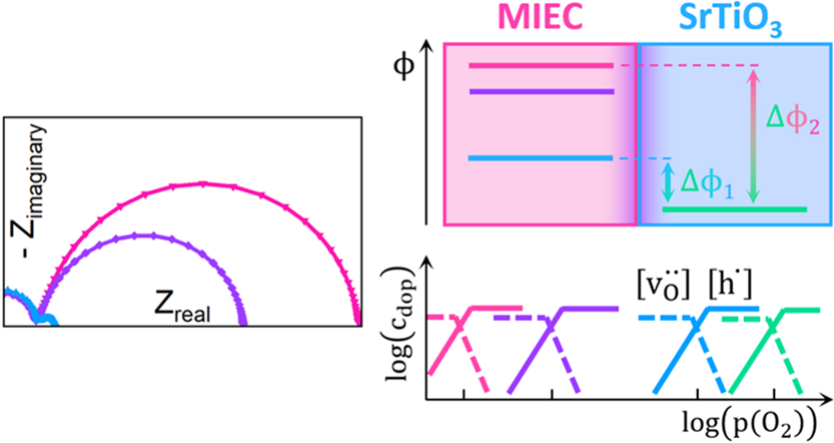

Mixed ionic and electronic conductors (MIECs) are a highly relevant material class in the field of solid-oxide cells and are, for example, promising candidates for electrodes with fast interfacial reaction kinetics. While there are many studies dealing with the bulk conductivities of such MIECs, models describing the interfaces between two mixed-conducting oxides have been far less developed. This study focuses on the investigation of space charges at the interfaces of the model perovskite SrTiO_3_ with different MIECs. Impedance spectroscopic measurements at 500 °C revealed that the MIECs under investigation can be divided into materials leading to negligible (YBa_2_Cu_3_O_7−δ_), moderate [(La,Sr)FeO_3−δ_, (La,Sr)CoO_3−δ_], and large [(La,Sr)MnO_3−δ_, (La,Sr)CrO_3−δ_] space charge resistances in SrTiO_3_ single crystals. The fundamental cause for these different space charge resistances is different space charge potentials, and we show that these can be determined by various methods with excellent agreement, ranging from X-ray photoelectron spectroscopy to impedance spectroscopy and photovoltage measurements. A model is introduced to correlate the ionic and electronic driving forces determining the space charges and to predict the space charge potentials from the electronic and ionic bulk properties of the corresponding mixed-conducting oxides. This model is also used to relate space charge potentials with reducibilities of MIECs, i.e., transition points from hole to vacancy compensation of an acceptor dopant in defect chemical Brouwer diagrams. The predicted trends are in good agreement with thermodynamic data on defect formation energies from the literature. Accordingly, the given model provides a widely applicable framework to predict and describe the space charge properties of a variety of MIEC heterojunctions.

## Introduction

Mixed ionic and electronic conductors (MIECs) are of high relevance in several fields of material science. For example, oxides with high electronic conductivity and moderate oxide ion conductivity, such as (La,Sr)(Co,Fe)O_3−δ_, are often used as cathodes in solid-oxide fuel cells.^1−5^ Mixed Li-ion and electron conductors, on the other hand, are common electrode materials in Li-ion batteries.^6−14^ Large band gap perovskite oxides, such as SrTiO_3_ and BaTiO_3_, constitute a further group of mixed conductors. Here, conduction may either be aimed at, e.g., in positive temperature coefficient resistors or memristors^15−18^ or is unwanted as in capacitors.^19−21^ Other examples of mixed-conducting oxides are Gd-doped ceria under reducing conditions^22,23^ or YBa_2_Cu_3_O_7−δ_ known for its superconductivity at proper oxygen vacancy concentrations.^24,25^

The bulk conductivities of such MIECs are very often studied, and for numerous oxides, more or less detailed Brouwer diagrams [defect concentrations in dependence of *p*(O_2_)] exist, e.g., SrTiO_3_ (STO),^26^ La_0.6_Sr_0.4_FeO_3−δ_ (LSF),^27^ or YBa_2_Cu_3_O_7−δ_ (YBCO).^24^ Also, space charge effects in ionic materials are frequently studied. A redistribution of ionic defects and formation of space charge layers are known to occur in pure ionic conductors, for example, at insulator|ionic conductor interfaces, grain boundaries, or ionic heterolayers.^28−30^ In MIECs, space charges at grain boundaries are accompanied by a redistribution of both ionic and electronic defects, which can lead to a wide variety of properties from blocking, as shown, for example, for SrTiO_3_^31,32^ or CeO_2_,^33,34^ to accelerating.^35−37^ Also space charges at free surfaces of SrTiO_3_ were studied in detail, e.g., by ^18^O tracer diffusion experiments,^38−40^ and models for relating the space charge potential to surface thermodynamics were developed.^41,42^ Moreover, different types of space charge effects in electrodes for solid-state batteries were described.^43^

Interestingly, space charge layers between two different MIECs are rarely considered in detail, even though they may affect the performance of the devices mentioned above. Space charges between two MIECs and mobile oxide ions may even play a functional role, for instance in high-temperature solid-oxide solar cells based on interfaces between SrTiO_3_ and other mixed ionic and electronic conductors.^44^ Experiments yielded photovoltages of over 1 V, and it is known that those photovoltages are related to space charges. However, in contrast to standard semiconductor physics, specific models describing the interplay of ionic and electronic defects in determining the corresponding space charges have not yet been developed yet. Accordingly, the following questions may be raised: What is the space charge potential between a large band gap MIEC, such as STO and a fuel cell MIEC electrode material, such as (La,Sr)FeO_3_ or (La,Sr)MnO_3_? How can we describe the thermodynamics of such space charges with two or more mobile charge carriers? How can we understand any differences found for different material combinations and experimental conditions?

In this study, we present impedance spectroscopic experiments at 500 °C as well as in situ near ambient pressure X-ray photoelectron spectroscopy (NAP-XPS) measurements, both revealing quantitative data on space charge potentials between nominally undoped STO single crystals and different highly electron- or hole-conducting MIECs, such as (La,Sr)FeO_3_ (LSF), (La,Sr)CoO_3_ (LSC), (La,Sr)MnO_3_ (LSM), (La,Sr)CrO_3_ (LSCr), and YBa_2_Cu_3_O_7_ (YBCO). In a second step, we develop a model in order to relate the space charge potentials to ionic and electronic properties of the materials, such as the valence band edge or the bulk oxygen vacancy concentration. Finally, we show that the reducibility of an MIEC oxide, i.e., the oxygen partial pressure where the electronic compensation of acceptor dopants (i.e., by electron holes) switches to ionic compensation (i.e., by oxygen vacancies), is a very good descriptor for the space charge potential induced by this MIEC in STO. Thus, in our study we develop the physicochemical basis for describing space charges between MIECs and for understanding the relation between space charge potentials and defect thermodynamics or Brouwer diagrams.

## Experimental Section

### Sample Preparation and Analysis

**Figure 1:**
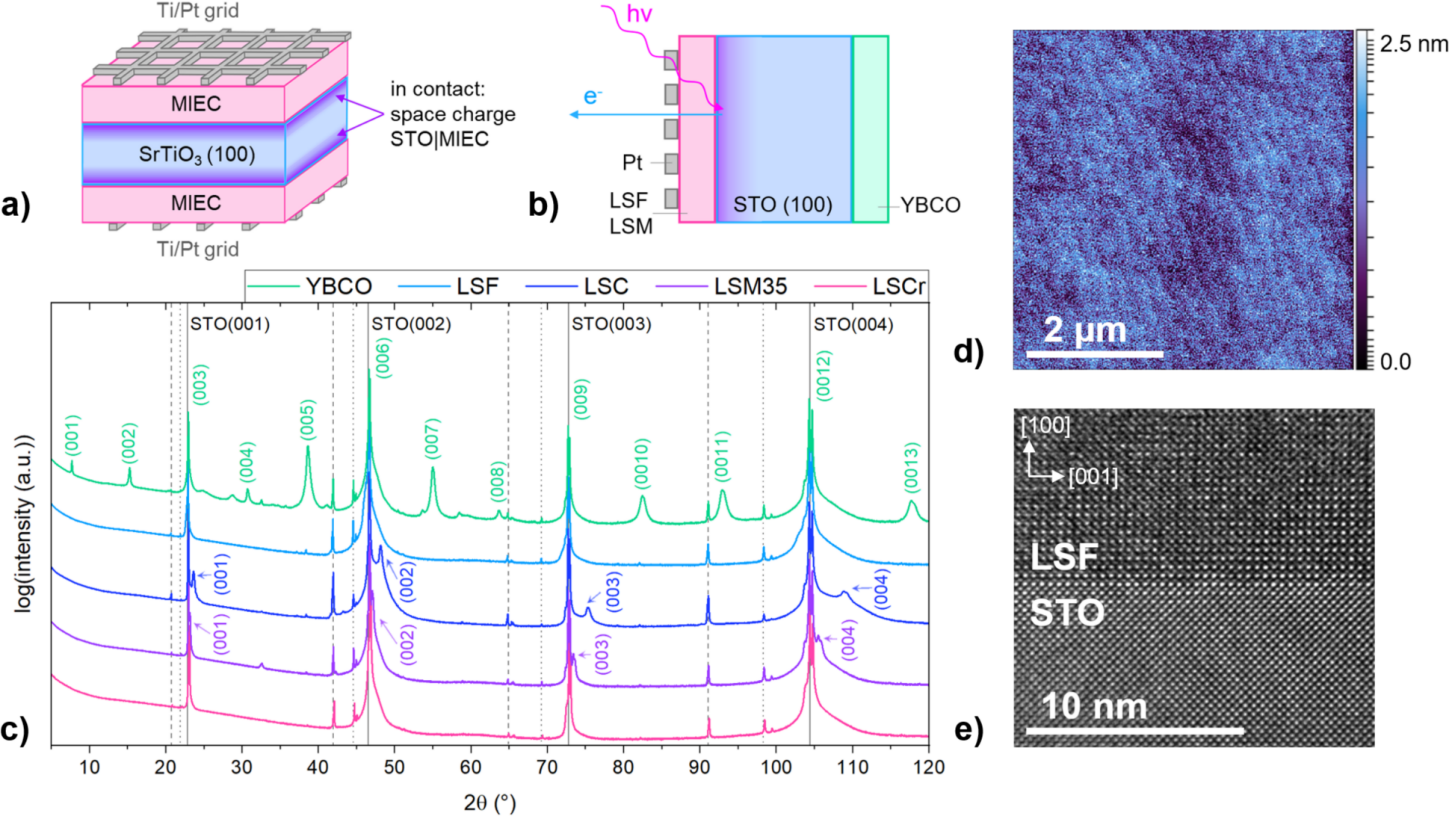
(a) Illustration of the sample geometry for impedance measurements, consisting of a STO (100) single crystal, onto which MIEC thin films and Ti/Pt current collector grids were symmetrically deposited. The purple highlighted region indicates the space charge zone between the STO and the MIEC investigated in this study. (b) Illustration of the sample geometry for NAP-XPS measurements, as well as a schematic illustration of the XPS measurement of the space charge region in STO being nearest to the interface. (c) θ – 2θ X-ray diffractograms of YBCO, LSF, LSC, LSM35, and LSCr thin films on STO (100), suggesting epitaxial growth of LSF, LSC, LSM, and LSCr. YBCO shows a preferentially oriented growth on STO. Spectral lines are marked through gray solid, dashed, and dotted lines representing Cu K_α1_ and K_α2_, K_β_, and W lines, respectively. (d) Exemplary AFM image of an LSF thin film grown on STO. (e) Bright-field TEM image of the LSF|STO interface, confirming epitaxial growth of LSF on STO.

Undoped STO (100) single crystals (10 mm × 10 mm × 0.5 mm, both sides polished) from Crystec (Germany) were used as substrates and cleaned with ethanol. MIEC thin films were symmetrically deposited onto both sides of these substrates by pulsed laser deposition (PLD) and thus acted as electrodes on SrTiO_3_. Table 1 summarizes the MIECs used here, and details of the PLD deposition parameters are given. For all impedance measurements, LSF, LSC, LSM35, and LSCr thin films with a thickness of approximately 50 nm were deposited, while the YBCO film thickness was approximately 100 nm. (Films were much thinner for XPS measurements, see details below.) Grid-shaped Pt current collectors of 100 nm thickness (15 μm stripes with 35 μm mesh distance) were applied on top of the MIEC films using lift-off photolithography and DC-magnetron sputtering (Baltec MED020, Leica Microsystems GmbH, Germany). Five nm Ti layers were deposited between the MIEC films and the Pt grids to ensure sufficient adhesion of the current collector. A sketch of the resulting sample geometry is given in Figure 1a; it also indicates the space charge regions in the STO investigated in this study.

**Table 1:** PLD Parameters Used for Depositing the MIEC Thin Films^a^

MIEC thin film	*p*(O_2_) (mbar)	temperature (°C)	fluence (J cm^–2^)
YBa_2_Cu_3_O_7−δ_ (YBCO)	3 × 10^–1^	800	1.5
La_0.6_Sr_0.4_FeO_3−δ_ (LSF)	4 × 10^–2^	600	1
La_0.6_Sr_0.4_CoO_3−δ_ (LSC)	4 × 10^–2^	550	1
La_0.65_Sr_0.35_MnO_3−δ_ (LSM35)	4 × 10^–2^	600	1
La_0.9_Sr_0.1_CrO_3−δ_ (LSCr)	1.5 × 10^–2^	700	1

a For all depositions, the target-substrate distance was set to 6 cm, the pulse frequency to 5 Hz, and the number of pulses to 4500.

The deposited MIEC thin films were characterized using X-ray diffraction (XRD), AFM, and TEM measurements. XRD measurements were conducted in the range of 5–120° in an Empyrean X-ray diffractometer (Malvern Panalytical, U.K.) in Bragg–Brentano geometry. AFM images were taken for all STO|MIEC sample surfaces with a Nanoscope V multimode setup (Bruker). Bright-field TEM measurements were conducted by cutting an electron-transparent focused ion beam (FIB) lamella from the interfacial region of the sample. Using standard lift-out techniques on a Thermo Fischer Scios 2 DualBeam FIB/SEM with a Ga-ion beam at 30 kV accelerating voltage, the FIB lamella was prepared and thinned, followed by a low-voltage cleaning step at 5 and 2 kV. TEM measurements were performed on a JEOL JEM-2100F field emission gun microscope with an image-side spherical aberration corrector and an acceleration voltage of 200 kV, as well as a Gatan Orisu SC1000 CCD camera to record the TEM images.

### Electrochemical Characterization

Impedance measurements were carried out using a Novocontrol Technologies Alpha-A high-performance frequency analyzer with a 4 wire impedance test interface. The samples were mounted into a sample tube by clamping between two quartz glass sheets enveloped in a fine Pt mesh. The sample tube was then slid into a furnace. All impedance measurements were performed in the frequency range of 1 MHz to 30 mHz with an applied AC_RMS_ voltage of 20 mV. The temperature during the measurements was set to 500 °C. The oxygen partial pressure *p*(O_2_) was varied between 1000 and 0.5 mbar using mixtures of gaseous O_2_ and N_2_, while the total pressure was held at atmospheric conditions. To ensure equilibration of the sample with the gas phase for each of the applied *p*(O_2_), the resistance of the sample was tracked. A change of less than 1.5% in sample resistance among four consecutive impedance measurements, i.e., within ca. 60 min, was considered sufficiently equilibrated. Typically, this state was reached after total equilibration times of more than 24 h. The impedance data in this equilibrium state were then used to calculate further parameters, such as the space charge potential and the space charge thickness.

### In Situ NAP-XPS Measurements

Samples for the in situ NAP-XPS measurements were prepared similar to the ones used for electrochemical measurements. Both sides of polished STO (100) single crystals (5 × mm 5 × mm 0.5 mm, Crystec) were cleaned using ethanol. YBCO (100 nm) was deposited via PLD onto one side of the crystal substrate. This layer enabled high absorbance during heating with an IR laser and acted as an electrode enabling temperature control via impedance spectroscopic measurements; see below. 4.2 nm of either LSF or LSM35 was deposited onto the other side. A quartz crystal microbalance was used to determine the exact deposited mass using the theoretical density from lattice parameters and thus the exact deposited film thickness of LSF and LSM. Circular Pt (20 nm) current collector grids with 3 mm diameter and a 25/50 μm strip width/mesh were applied onto the LSF and LSM thin films using photolithography and sputtering.

In the XPS chamber, a customized sample holder with a 4.5 × 4.5 mm hole for laser heating with a near-infrared diode laser was used. Electrical contact was established by the use of Pt/Ir wires that contacted the current collector grid and the YBCO counter electrode. A sketch of this sample type and the corresponding measurements are given in Figure 1b. A detailed description of the setup is published elsewhere.^45^ By means of in situ impedance measurements, the temperature was calculated from the measured STO bulk resistance and compared with known conductivity data, enabling temperature control of the sample. In situ NAP-XPS measurements were carried out at 500 °C at a *p*(O_2_) of 1 mbar. These NAP-XPS measurements were conducted in a lab-based setup with a PHOIBOS NAP photoelectron analyzer (SPECS, Germany) and a monochromated Al K-α XR 50 MF microfocus X-ray source, and detailed scans were acquired at an analyzer pass energy of 30 eV. Additional XPS calibration measurements were performed on a 0.5 wt % Nb-doped STO (100) crystal (5 mm × 5 mm × 0.5 mm, Crystec) in reducing conditions (0.1 mbar H_2_) at the same temperature. Data analysis was conducted with CasaXPS software.

## Results

### Sample Characterization

Measured diffractograms are shown in Figure 1c. For all measured samples, the STO substrate reflections at 22.9, 46.6, 72.7, and 104.3°, assigned to the STO (001), (002), (003), and (004) reflections, as well as the corresponding spectral lines, are the most prominent reflections. LSF and LSCr reflections overlap with the substrate reflections; therefore, no additional thin film reflections are visible in the diffractogram, indicating epitaxial growth of the thin films, as already shown in earlier studies.^44,46^ For LSM35, additional reflections are detectable, which are closely positioned next to the substrate reflections at 23.14, 47.0, 73.4, and 105.5°. These reflections are assigned to the LSM (001), (002), (003), and (004) reflections, indicating a thin film growth in the preferred STO (001) direction and thus the epitaxy.^47,48^ Compared with other MIEC thin films, LSM35 exhibits a small reflection at 32.4°, which cannot be definitely identified. Likely, it is either another orientation of LSM (110) or a secondary phase, such as SrO. However, due to its low intensity compared to the (001), (002), (003), and (004) reflections, a preferential thin film growth in the (001) direction of LSM35 on STO can be assumed.

The same can be found for LSC, where the thin film (001), (002), (003), and (004) reflections are found in close proximity to the substrate reflections at 23.6, 48.1, 75.3, and 108.9°, respectively.^49^ In contrast to the other cubic perovskite-type MIEC thin films, YBCO exhibits an orthorhombic crystal structure. As a consequence, the diffractogram shows more reflections. However, besides small impurities that were not clearly assignable, the YBCO pattern also suggests preferential thin film growth in the (001) direction. This is also in accordance with earlier studies.^50,51^

Figure 1d displays, by way of example, an AFM image of the LSF surface. The root-mean-square (RMS) roughness of the ca. 30 nm thin film is 283.5 pm, indicating a very smooth surface. Moreover, terraces originating from the STO single crystal beneath the ca. 30 nm thin film are detectable. The same LSF sample was used for TEM measurements. Figure 1e shows a TEM image of the LSF|STO interface, confirming epitaxial growth of the thin film, as the atomic array in the LSF matches with the array of the STO atoms. Earlier TEM studies on LSC|STO and LSM|STO also confirmed epitaxy of the thin films.^49,52^

### Impedance Spectroscopic Measurements

**Figure 2:**
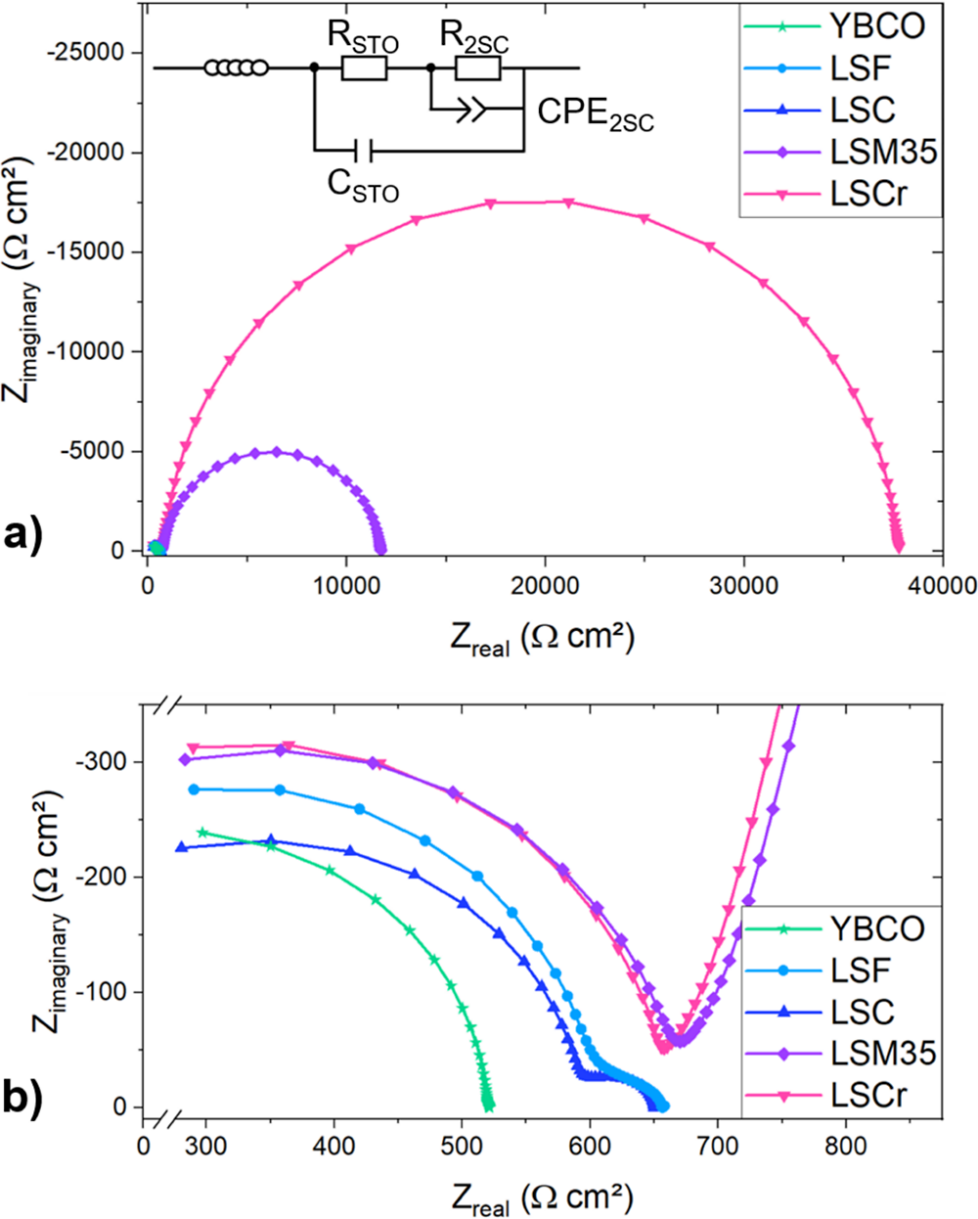
Nyquist plot of the measured impedance spectra for various MIECs on STO at 500 °C and 200 mbar of *p*(O_2_). (a) Full Nyquist plots. (b) Inset of (a).

Figure 2 shows Nyquist plots measured at 200 mbar of *p*(O_2_) and 500 °C for various MIECs on STO including a closeup of the high-frequency region. Most impedance spectra display two features in the entire *p*(O_2_) range under investigation (1 bar to 5 × 10^–4^ bar). The high-frequency arc is identified as the STO single-crystal bulk feature, i.e., the bulk transport resistance parallel to the dielectric capacitance. This bulk feature displays a similar size for all samples, which is expected for nominally identical single-crystal substrates at the same temperatures. The corresponding bulk conductivity fits excellently to the expected hole conductivity of such undoped STO single crystals,^26^ indicating predominantly electronic conduction. From the parallel capacitance feature and the sample geometry, a relative permittivity of 150–160 at 500 °C was extracted, which is a typical value for STO.

The low-frequency feature is assigned to the interfacial space charges at the two MIEC|STO interfaces, in which positive mobile charge carriers, i.e., holes and oxygen vacancies in STO are depleted. The main argument for this assignment is the absolute value of the capacitance, which fits well with the expected values for space charges; see below. Additionally, in the given *p*(O_2_) range, primarily hole conduction is expected in the MIEC thin films in accordance with its defect chemical models.^27,53−55^ Hence, ionic resistances, such as the oxygen exchange surface reaction, are largely irrelevant and not expected to cause additional arcs. A more detailed discussion of these space charge interfaces is given below. The impedance spectra of STO|YBCO do not show any apparent space charge feature. Consequently, it is assumed that any hole depletion in the space charge between STO and YBCO is either absent or too small to be detected by our impedance measurements. Otherwise LSF and LSC on STO exhibit the smallest space charge semicircle, followed by LSM35, where the space charge feature is already very prominent. LSCr causes the most pronounced space charge arc.

The impedance data were fitted using the equivalent circuit in Figure 2a, where *R*_STO_ and *C*_STO_ are the mixed ionic and electronic resistances and the dielectric capacitance of the STO single crystal, respectively. *R*_2SC_ and *C*_2SC_ are the resistance and capacitance of the space charges at the two STO|MIEC interfaces, respectively. Since the measured samples are symmetrical, and thus identical space charges are expected at both STO|MIEC interfaces, the fitted resistance was divided by a factor of 2 in order to obtain the resistance of a single space charge *R*_SC_. Equally, *C*_2SC_ was multiplied by a factor of 2 to obtain the capacitance of one space charge *C*_SC_. Please note that a nested circuit instead of two serial RC elements was used. This circuit takes better account of the fact that actually the capacitive counter charge of the interfacial space charge in STO and the charge of the geometrical sample capacitor are located in one and the same MIEC layer.^56^ This circuit also leads to more consistent space charge capacitances compared to fits using two serial RC elements: while differences are almost negligible for large space charges (LSM, LSCr), the two serial RC models lead to significantly lower *C*_SC_ values for LSC and LSF (by even 30% at low *p*(O_2_), where the space charge feature is only visible as a small shoulder of the STO bulk arc in the Nyquist plot). Moreover, in order to improve the fit quality, a constant phase element (CPE) with an impedance of  instead of a capacitor was used for the space charge. The space charge capacitance was then calculated according to .^57^ Typical capacitance values are about 450 nF/cm^2^ for LSF and LSC, 750 nF/cm^2^ for LSM and 670 nF/cm^2^ for LSCr at 500 °C and 200 mbar.

**Figure 3:**
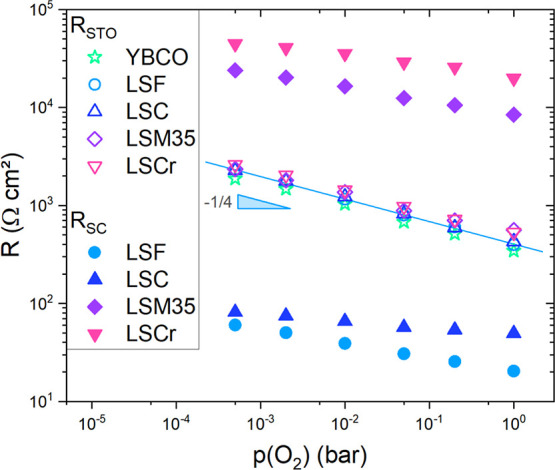
Plot of the fitted bulk resistance values of STO *R*_STO_ and the space charge feature *R*_SC_ at 500 °C, all normalized to the sample area. *R*_STO_ exhibits a slope of , which is in accordance with STO defect chemical models, i.e., Brouwer diagrams. The slopes for *R*_SC_ vary between  and . Most important is the pronounced difference between the *R*_SC_ values of LSF/LSC and LSM/LSCr.

Figure 3 shows the fitted resistance values for the STO bulk and space charge features. As already mentioned, the resistance values for all STO features do not vary significantly as nominally identical STO single crystals were used as substrates. Furthermore, the *p*(O_2_) dependency of the STO resistances exhibits a slope of  in log–log plots. This corresponds to the slope of hole conductivity expected from the Brouwer diagram of slightly acceptor-doped STO in the given *p*(O_2_) range.^26^ (Please note that nominally undoped SrTi O_3_ single crystals are effectively slightly acceptor-doped. In our case, we can assume cation vacancies corresponding to ca. 24 ppm singly charged acceptor dopants.^26^)

The resistances of the space charge features vary by several orders of magnitude. While the area-specific resistance of LSF and LSC shows values in the range of 10^1^–10^2^ Ωcm^2^, the resistances of LSM and LSCr are in the range of 10^4^–10^5^ Ωcm^2^. In contrast to the  slope of the STO bulk resistance with increasing oxygen partial pressure, the slopes of the space charge resistances are between  and . Actually, the *p*(O_2_) dependence of *R*_SC_ is expected to reflect the difference of the bulk *p*(O_2_) dependencies of the two individual materials; see data analysis. Thus, the exact value of the slope is expected to vary with *p*(O_2_). A more detailed analysis, however, would require data in a broader *p*(O_2_) range and will be given in a forthcoming work.

### Space Charge Potential

In principle, the interfacial space charge at the MIEC|STO contact has two contributions, one from STO and one from the MIEC. However, since all MIECs leading to measurable space charge features exhibit very high concentrations of electronic charge carriers due to their high acceptor doping, their own space charge zones are negligibly thin, and thus the entire relevant space charge region is assumed to be in STO, see also below. Depending on the relation of the mean free path of electronic charge carriers to the space charge thickness, either a thermionic emission or a drift-diffusion model has to be used to describe the space charge resistance. Due to the comparatively low mobility of holes in the order of 0.1 cm^2^/(Vs), determined from Hall conductivity measurements,^58^ multiple scatter events are expected within the space charge zone. More specific, the mean free path of electronic charge carriers can be estimated from the experimental mobility and the effective mass when assuming thermal velocity.^59^ For the hole mobility in SrTiO_3_ at 500 °C^58^ and an effective hole mass in the range of a few electron masses, we get mean free paths in the sub nm range. Hence, the very thick space charges found here (above 100 nm) suggest the validity of the drift-diffusion model. Also, tunneling does not have to be considered for such thick space charges. In the literature,^60^ it was shown that in the case of a drift-diffusion model and a Schottky approximation, the area-specific bulk resistance *R*_STO_ and the area-specific resistance of one space charge *R*_SC_ of STO can be used to calculate the space charge potential Δϕ in STO according to eq 1
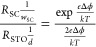
1where *d* is the thickness of the single crystal, *e* is the elementary charge, *k* is the Boltzmann constant, and *T* is the temperature. In this Schottky approximation, the space charge thickness *w*_SC_ is given by
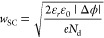
2where ε_0_ is the vacuum permittivity, ε_*r*_ is the relative permittivity of STO, and *N*_d_ is the dopant concentration. For the following calculations of Δϕ and *w*_SC_, ε_r_ was assumed to be constant throughout the STO single crystal, including the space charge area.

**Figure 4:**
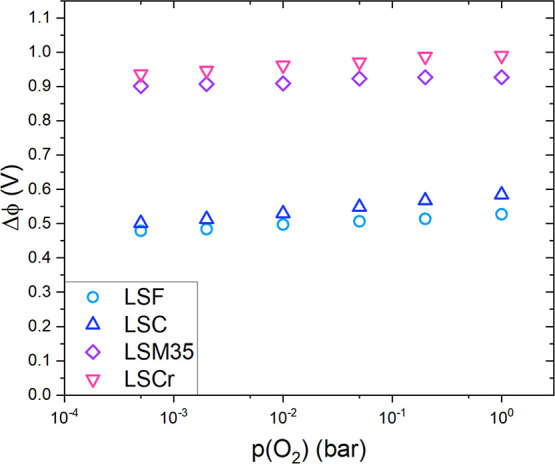
Plot of the space charge potential Δϕ, calculated from eqs 1 and 2 for 24 ppm singly charged acceptor dopants^61^ against the oxygen partial pressure *p*(O_2_). The plotted space charge potentials are shown for the space charge regions between STO and LSF, LSC, LSM35, or LSCr.

Eq 2 again indicates that very thin space charge zones (≪1 nm) can be expected in the highly Sr-doped MIECs. Hence, Δϕ of the space charge in STO is almost identical to the difference between the electrostatic potentials in the MIEC and STO bulk, i.e, Δϕ ≈ ϕ^MIEC^ – ϕ^STO^, at least when neglecting any additional interfacial dipole layer at the core of the interface. For known dopant concentrations, eqs 1 and 2 can be used to calculate Δϕ. Measurements of nominally identical STO single crystals revealed a dopant concentration of 24 ppm singly charged acceptor dopants.^26^ This *N*_d_ value was used, and numerically solving eqs 1 and 2 then leads to the space charge potentials for the space charge zones between STO and LSF, LSC, LSM, or LSCr as shown in Figure 4. The same trend as in the impedance plots is again present, i.e., the space charge potential shows moderate values of 518 mV for LSF and 590 mV for LSC. For LSM35, Δϕ is much higher and equals 916 mV. The highest Δϕ was calculated for LSCr with a value of 992 mV, all at 1 bar O_2_. These values are averaged over two to three samples. Typical fit errors of *R*_STO_ are <1% for all samples. Typical fit errors of the space charge resistances for LSF and LSC are around 1.5% at higher *p*(O_2_) and increase up to 5% at 0.5 mbar. The fit errors of *R*_SC_ for LSM and LSCr are <1% throughout the measured *p*(O_2_) range. This transfers to a fit error of Δϕ in the 1% range. Standard deviations of Δϕ for several nominally identical samples are 20 mV for LSF and LSC, 12 mV for LSM35, and 10 mV for LSCr. For all investigated space charge zones, Δϕ slightly decreases with a decrease in the oxygen partial pressure. However, a detailed discussion of this partial pressure dependence of Δϕ is beyond the scope of this work; see also above.

Alternatively, the space charge thickness *w*_SC_ could also be obtained from the fit results of *C*_SC_, assuming bulk permittivity (from *C*_STO_) in the space charge zone. Table 2 summarizes the *w*_SC_ values determined from impedance spectra in 200 mbar O_2_ at 500 °C. For the space charge arcs of LSCr and LSM, we obtain a very good agreement with the *w*_SC_ values calculated from Δϕ and 24 ppm negative elementary charges as dopants in a unit cell. For LSF and LSC, fit values are higher than those obtained from eq 2. However, here we have to take into account that the space charge arcs of LSF and LSC not only overlap with the STO arc but also are much smaller and only visible as small shoulders of the main arc, in contrast to the larger and well separated LSM and LSCr space charge arcs. This impairs the accurate fit analysis of the impedance spectra. At 200 mbar, for example, fit errors of *Q* are around 2% for LSM and LSCr. For LSF and LSC, fit errors of *Q* are around 5% in 1 bar O_2_, and they increase up to 30% for 0.5 mbar. Assumingly, this is the main reason behind the discrepancies of *w*_SC_ for the materials. Most probably the *w*_SC_ values from Δϕ are more accurate since according to eq 1, errors in *R*_SC_ are less critical than those for , with *A* being the area.

**Table 2:** Comparison of the Space Charge Layer Thicknesses at 200 mbar O_2_ Calculated through the Capacitance of the Space Charge C_SC_ and the Space Charge Potential Δϕ

MIEC thin film	*w*_SC_ from Δϕ and eq 2 (nm)	*w*_SC_ from *C*_SC_ (nm)
LSF	140	347
LSC	147	276
LSM35	190	184
LSCr	200	222

### XPS Measurements and Comparison with Photovoltages

In addition to the space charge potentials extracted by impedance spectroscopy, the space charge zones were investigated using NAP-XPS. The XPS experiments were performed on STO (100) single-crystal interfaces covered by 4 nm thin MIEC films (LSF and LSM to represent one material with a moderate and one with a very large space charge potential, respectively) at 500 °C in an oxygen partial pressure of 1 mbar. A 4 nm thin film is still >10 times thicker than the Debye length and thus is expected to exhibit bulklike behavior while nevertheless being thin enough to allow collection of photoelectrons from the parts of the STO space charge region being nearest to the MIEC|STO interface. Approximately, 10% of the resulting photoelectrons in STO were able to leave the sample.

These measurements utilize the fact that the measured “binding energy” in XPS is actually the energy difference between the respective core level and the Fermi energy. Within a space charge zone, band bending (induced by the leveling of Fermi energies, *E*_F_) leads to an apparent shift of the core level binding energies. The inelastic mean free path of photoelectrons is 1.6–2 nm and thus 100 times smaller than the width of the space charge zone. Thus, the detected electrons from STO indeed represent a region in the space charge with a potential being close to the full Δϕ (vs STO bulk). In weakly doped semiconductors, a shift of the measured binding energy is thus proportional to the space charge potential. Accordingly, in our case, the Ti 2p_3/2_ peak position is indicative of the band bending at the interfacial end of the space charge zone in STO. The Ti 2p_3/2_ peak was chosen for metrological reasons as Ti is not present in the investigated MIECs, and thus the measured electrons come unambiguously from the STO single crystal, i.e., the space charge region. Additionally, the Ti 2p_3/2_ peak exhibits the highest intensity of all measurable Ti peaks. However, for quantitative interpretation, a reference measurement with a known Fermi level position is required. Since space charges also occur on native STO surfaces,^39^ reference with Δϕ = 0 cannot be taken from undoped STO.

**Figure 5:**
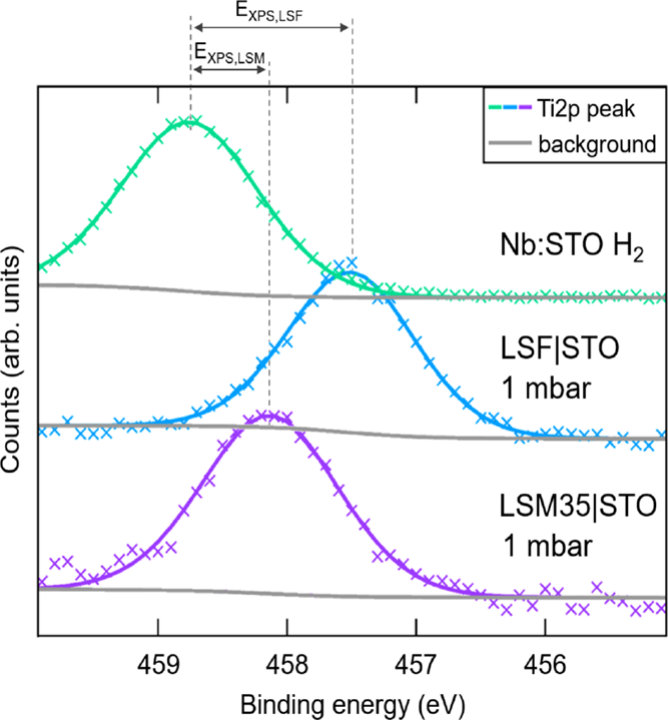
In situ measured binding energies of the Ti 2p peak for the junctions LSF|STO and LSM35|STO at 500 °C in an oxygen partial pressure of 1 mbar. An Nb-doped STO single crystal was used as a reference. *E*_XPS_ expresses the binding energy difference between the Nb:STO peak and the LSF or LSM peak.

As an energy reference, a 0.5 wt % Nb:STO (100) single crystal in H_2_ at 500 °C was used. Due to the high donor doping level, the Fermi level of Nb:STO E_F,Nb:STO_ is very close to the conduction band edge and has a very small and narrow surface space charge, especially under reducing conditions. Hence, it can serve as an energy reference, where the Fermi energy can be approximated by the conduction band edge in the bulk, provided the surface potential due to surface dipoles does not differ between undoped STO and Nb:STO of the same orientation. The Ti 2p_3/2_ peaks of a Nb:STO single crystal as well as nominally undoped STO single crystals with LSF and LSM top layers are shown in Figure 5. Clearly visible, the binding energies for all three samples are different. The Ti 2p peak of LSF at 1 mbar was detected at 457.51 eV, whereas the Ti 2p peak of LSM was observed at 458.04 eV. This measurable shift of the Ti 2p binding energies is attributed to the different space charges.

**Figure 6:**
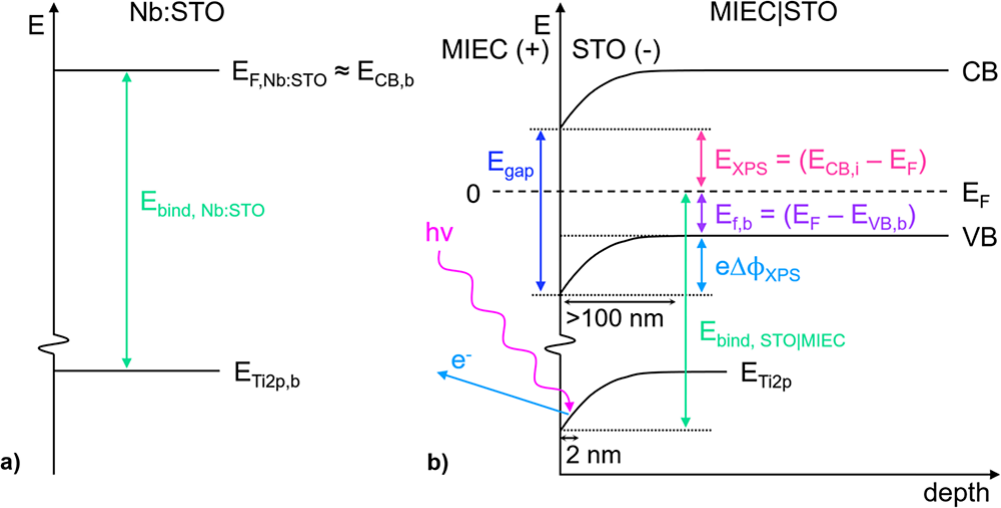
(a) Schematic illustration of the energy bands in Nb:STO, where no band bending is expected. (b) Schematic illustration of the Ti 2p band bending at the MIEC|STO interface, where photo electrons are ejected from the surface nearest region. Illustrated is also the STO band gap with parameters needed for calculation of the space charge potential from XPS measurements Δϕ_XPS_. *E*_gap_ is the band gap energy, *E*_XPS_ is the energy difference between the conduction band edge and the measured Ti 2p binding energy, and *E*_f,b_ is the energy difference between the valence band edge and the Fermi level in STO. The polarity of the space charge is indicated with (−) for the negative excess charge in STO and (+) for the positive excess charge in the MIEC.

For space charge determination, we will focus on the energy difference between the Nb:STO surface and the LSF|STO or LSM|STO interface, shown as *E*_XPS_ in Figure 5. As we assume that for Nb:STO, the surface Fermi energy is at the bulk conduction band minimum, the binding energy difference E_XPS_ reflects the Fermi energy at the undoped STO|MIEC interface, relative to the conduction band minimum, as sketched in Figure 6. This can be understood from the fact that the binding energy is measured with respect to the (bulk) Fermi level *E*_F_, and this distance differs for the different MIECs due to different band bending. This leads to the situation sketched in Figure 6b: The binding energy difference *E*_XPS_ measured between Nb:STO and STO with MIEC reflects the difference between the bulk Fermi level of undoped STO and the conduction band at the STO|MIEC interface due to eq 3
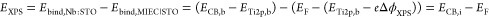
3

**Figure 7:**
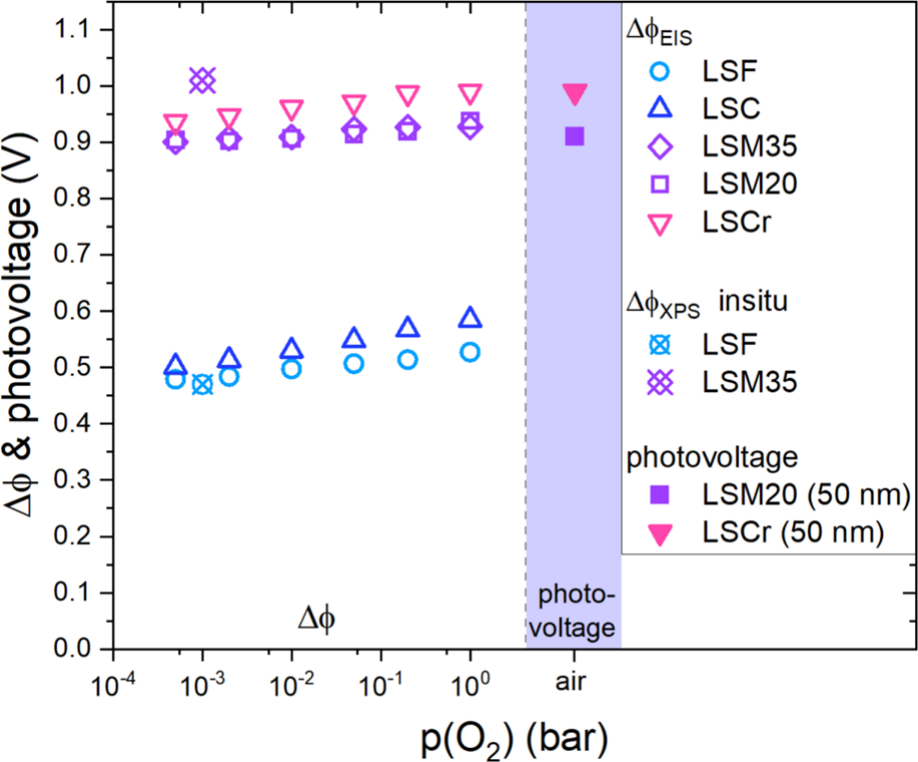
Plot of the space charge potential extracted from impedance data Δϕ_EIS_, the space charge potential obtained through in situ XPS measurements Δϕ_XPS_, and photovoltages produced by nominally identical junctions under UV illumination^44^ against the oxygen partial pressure *p*(O_2_). The plotted space charge potentials are shown for the space charge regions between STO and LSF, LSC, LSM35, LSM20, or LSCr.

Since doping of 0.5% does not cause a chemical shift for Nb:STO compared to undoped STO, the distance between the energy of the conduction band in the bulk *E*_CB,b_ and the energy of the Ti 2p_3/2_ band in the bulk *E*_Ti2p,b_ is the same for both Nb:STO and undoped STO. The energies of the conduction band in the bulk (*E*_CB,b_) and at the interface (*E*_CB,i_) differ by *e*Δϕ_XPS_ (eq 4), i.e., we find
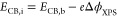
4The distance of the Fermi level to the valence band in the bulk
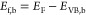
5is given by the defect chemistry of undoped STO (eq 5). Thus, we get (eq 6)
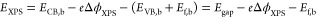
6and finally (eq 7)
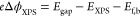
7*E*_f,b_ was calculated in accordance with the defect model of such nominally undoped STO single crystals^26^ through the relation , where *c*_VB_ is the effective density of states for undoped STO.^62,63^ At 500 °C in 1 mbar O_2_, a value of 0.83 eV was found. *E*_gap_ = 2.80 eV at 500 °C^62^ was used, and the resulting Δϕ_XPS_ values are shown in Figure 7 and are plotted next to space charge potentials Δϕ_EIS_ extracted from impedance measurements in dependence of *p*(O_2_). In general, the values for Δϕ_XPS_ and Δϕ_EIS_ show good agreement. Δϕ_XPS,LSF_ exhibits a value of 0.47 V at 1 mbar *p*(O_2_) compared to 0.48 V for Δϕ_EIS,LSF_ at 2 mbar *p*(O_2_). Δϕ_XPS, LSM35_ yields 1.01 V at 1 mbar *p*(O_2_) compared to Δϕ_EIS,LSM35_ with 0.91 V at 2 mbar *p*(O_2_). (For the XPS measurements, we suppose an uncertainty of a few 10 mV from our model approximations.) The very moderate deviations between Δϕ_XPS_ and Δϕ_EIS_ could be either attributed to small variations between samples or the assumptions and approximations made for each measurement approach. The difference of 540 mV between Δϕ_XPS,LSF_ and Δϕ_XPS,LSM35_ at 1 mbar *p*(O_2_) is also in good agreement with the Δϕ_EIS_ difference of 423 mV between LSF and LSM35 at 2 mbar *p*(O_2_).

In addition to the Δϕ_XPS_ values, Figure 7 also presents open-circuit photovoltages measured for nominally identical LSM20|STO (La_0.8_ Sr_0.2_ Mn O_3_) and LSCr|STO junctions upon UV illumination. The photovoltages are taken from the literature^44^ and were measured on very similar samples (same STO single crystals, same PLD procedure); typical absorption depths of the corresponding UV light are 2 μm.^44,64,65^ The measured photovoltages are plotted in the purple shaded area of the plot. For an LSM20 and LSCr thin film of 50 nm thickness, photovoltages of 0.91 and 0.99 V, respectively, were measured. For comparison, additional impedance measurements were performed also for LSM20 thin films on STO single crystals, which were deposited under the same conditions as the LSM35 thin films (Table 1) with the same sample geometry; see Figure 1a. The resulting space charges are very similar to those of LSM35. The photovoltages are thus also in good agreement with the Δϕ_EIS_ values for LSM and LSCr. This is to be expected as photovoltages fundamentally rely on the space charges at material junctions. Incident UV rays generate electron–hole pairs by exciting electrons into the conduction band and thereby collapsing the space charge. As a result, the upper limit of the measured photovoltage is given by the space charge potential. Overall, we can thus conclude that several fundamentally different methods, EIS, XPS, and photovoltage measurements under UV illumination, predict space charge potentials with very good quantitative agreement. In the following, a defect chemical model is derived to describe and understand these space charge potentials and their dependence on MIEC properties.

## Discussion

### Model Considerations for the Ion-Electron Interplay in the Space Charge Zone

**Figure 8:**
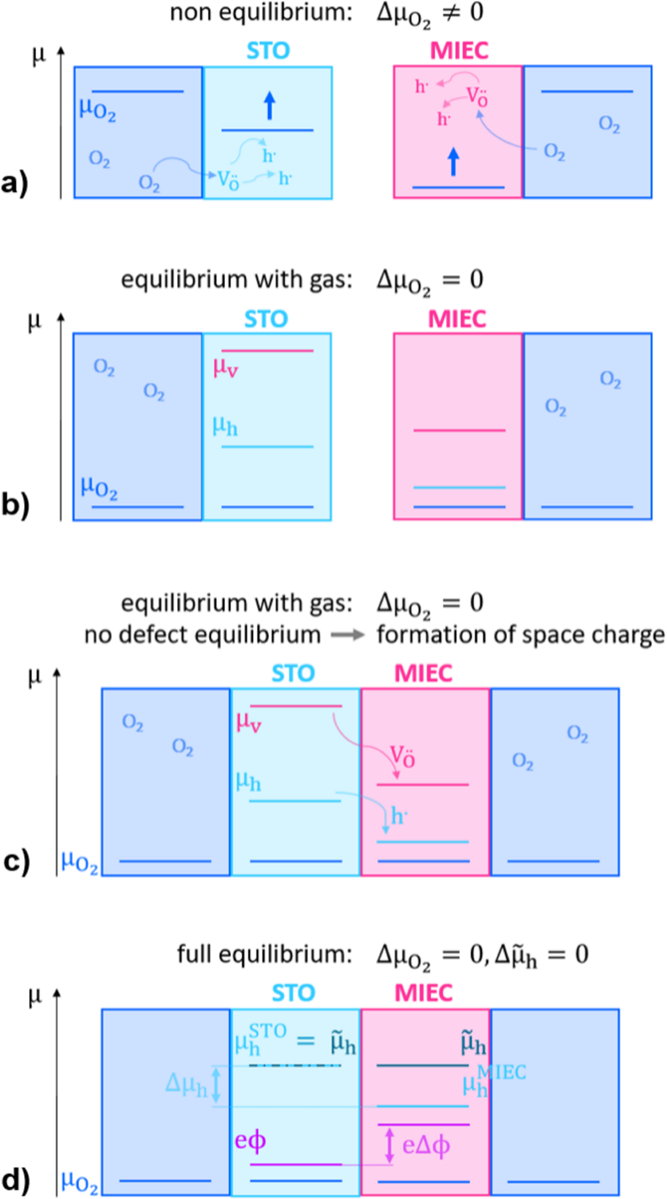
Schematic illustration of the model considerations regarding equilibrium with the *p*(O_2_) atmosphere and space charge formation. (a) Equilibration of  and  with  through oxygen in- or excorporation. (b) Alignment of μ_h_ and μ_v_ in the equilibrated STO and MIEC, while not being in contact. (c) Once in contact, a space charge region between STO and MIEC forms through charge transfer to achieve defect equilibrium. (d) For equilibrium with  and defect equilibrium, i.e., full equilibrium where  and  are 0, the space charge potential Δϕ can be described either through Δμ_h_ or Δμ_v_. Here Δμ_h_ is shown. ϕ is arbitrarily set to 0 in the STO bulk. Different absolute scales of μ_h_, μ_v_, and μ_O_2__ are used in these sketches.

For a given normalized oxygen partial pressure *p*(O_2_), an atmospheric oxygen chemical potential  can be defined as eq 8
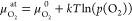
8with the standard chemical potential . If a sample with sufficiently fast oxygen exchange kinetics is exposed to a certain *p*(O_2_) and thus  at an elevated temperature, its oxygen chemical potential  starts to align with  until . For samples consisting of multiple materials, such as STO|MIEC, μ_O_2__ of each material, i.e.,  and , aligns with  until an equilibrium is reached according to eq 9
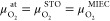
9This is schematically shown in Figure 8a. In order to achieve equilibrium with the applied *p*(O_2_), oxygen has to be incorporated or released from STO and the MIEC. For each oxygen atom incorporated, an oxygen vacancy  is consumed, and to ensure charge neutrality, two holes h^·^ have to be created. The defect chemical reaction can thus be written as eq 10
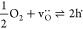
10In equilibrium, the chemical potentials of gaseous oxygen, oxygen vacancies, and electron holes are related by the respective chemical potentials via
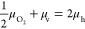
11Based on this consideration, μ_O_2__ in both MIEC and STO is defined by the chemical potentials of holes and vacancies. The equilibrium condition (eq 9) and eq 11 yield for any difference Δ between MIEC and STO
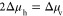
12meaning that, if both materials equilibrate in the same *p*(O_2_) atmosphere, the chemical potentials of the holes and vacancies are not the same but tied to each other by the chemical potential of neutral oxygen, which is the same in both materials, even if they are not yet in contact.

**Figure 9:**
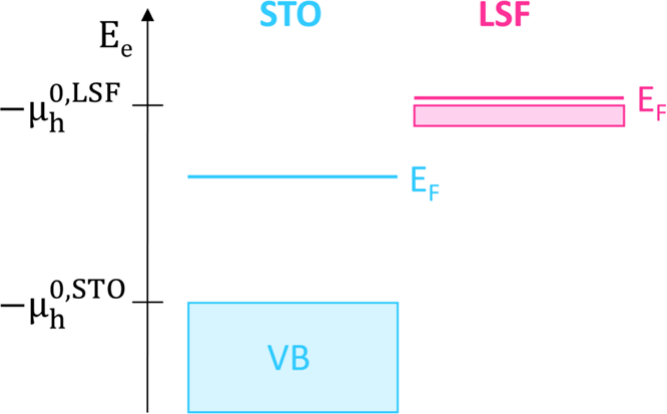
Sketched band scheme of STO and LSF before contacting, indicating that the different Fermi levels *E*_F_ = −μ_h_ are strongly related to the very different  values.  is −*E*_VB_ for STO and represents the hole polaron level for LSF.

We first consider STO and the MIEC separately (i.e., without being in contact). Then, the specific oxygen vacancy and hole concentration are determined by doping, temperature, and partial pressure in accordance with the respective Brouwer diagram. For two different materials in equilibrium with O_2_, we thus have two different oxygen vacancies and hole concentrations and different defect chemical potentials. Accordingly, the defect equilibrium between the two phases is not given yet. This is sketched in Figure 8b. If we now bring STO and MIEC into contact, not only the formally neutral oxygen but also each defect species needs to be in equilibrium. This requires a transfer of charged defects and thus induces an electrostatic potential difference, which is sketched in Figure 8c. We thus have to extend the chemical potential definition by an electrostatic term, leading to the electrochemical potential μ̃ = μ + *ze*ϕ. In dilute situations with normalized concentration *c* and charge number *z,* we find μ̃ = μ0 + *kT* ln(*c*) + *ze*ϕ. As a consequence, we may write the equilibrium condition as
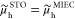
13
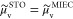
14We first focus on the holes, and as in the case of a contact between two pure semiconductors, we get from the equilibrium condition for holes (eq 13) the relation
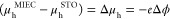
15see Figure 8d. Including site restriction for holes in the highly doped MIEC and a dilute situation for STO this would transform to
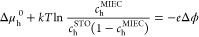
16This is also sketched in Figure 9 for the STO|LSF contact and a LSF polaronic level at .

**Figure 10:**
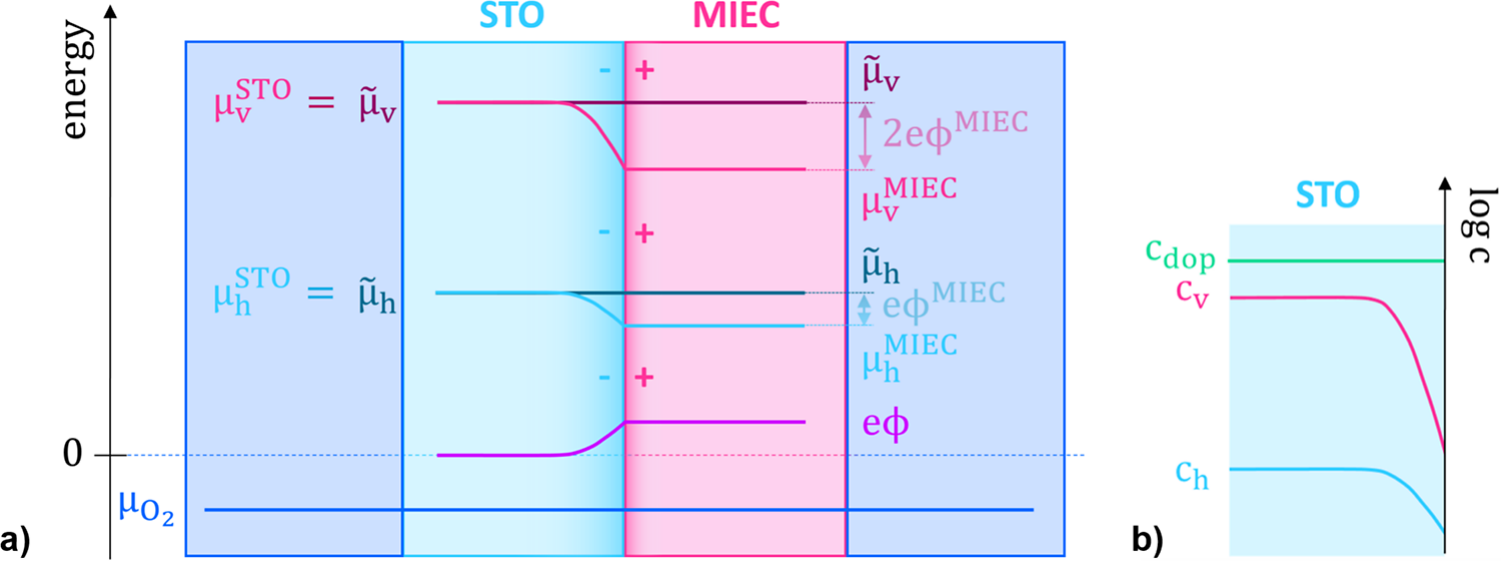
Schematic illustration of the space charge region at the STO|MIEC interface in equilibrium with surrounding *p*(O_2_). (a) Behavior of μ_h_, μ_v_, and *e*ϕ in the interfacial region. ϕ is arbitrarily set to 0 in the STO bulk. The polarity of the space charge is indicated with (−) for the negative excess charge in STO and (+) for the positive excess charge in the MIEC. (b) Schematic illustration of the depletion region in STO according to the Mott–Schottky model, where the dopant concentration *c*_dop_ is constant throughout the STO and the defect concentration of holes *c*_h_ and vacancies *c*_v_ depletes toward the interface.

The same considerations can be made for oxygen vacancies, and similar to a space charge between two ionic conductors, we find from the equilibrium condition for vacancies (eq 14)
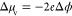
17In general, Δϕ is the equilibrium electrostatic potential difference between the two considered mixed ionic and electronic conductors; here, STO or LSF, LSC, LSM and LSCr. In our case, the space charge region is localized predominantly in STO, and thus Δϕ equals the space charge potential in STO, as schematically shown in Figure 10a. In this figure, we also sketched the chemical and electrochemical potentials of all particles on one and the same “absolute” scale. The position for μ_O_2__ corresponds to the standard Gibbs energy of O_2_.^66^ The position of  is estimated from  with  being the Fermi level *E*_F_ of STO and thus reflecting the work function of STO vs vacuum.^67^ This, however, is only a rough estimate since work functions also include surface potential terms which may strongly differ from our STO|MIEC interfaces.  finally results from eq 11. Please note that thermodynamic data from DFT calculations usually only consider neutral vacancies and do not split this into the two contributions of charged vacancies () and holes (). At a first glance, this seems to indicate a conflict since one and the same space charge has to fulfill two conditions, eqs 15 and 17. This is only the case if
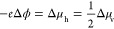
18holds. This, however, is a necessary requirement of equilibrium with the gas phase. From eq 11, we find , and for Δμ_O_2__ = 0, eq 18 is automatically fulfilled. Thus, in equilibrium with the gas phase, both holes and vacancies simply need the same space charge potential Δϕ to get into equilibrium. As a consequence, the potential between two mixed ionic and electronic conductors is sufficiently described by considering only one charge carrier species, and as in equilibrium, ionic and electronic charge carriers are anyway interconnected through Δμ_v_ and Δμ_h_, respectively, as given in eq 12. For predicting equilibrium space charge potentials, one may thus consider the defect for which most information is available. In our specific case, the sign of the space charge potential (Δϕ > 0, i.e., ϕ is more positive in the MIEC) also indicates that both μ_v_ and μ_h_ are higher in the bulk of STO, and thus the Fermi level *E*_F_ = −μ_h_ is lower in STO before contacting.

Since the chemical potential is directly related to the defect concentrations, the decrease of the chemical potential in the STO space charge region toward the STO|MIEC interface is reflected in the depletion of the corresponding defect concentration toward the interface. This is schematically shown in Figure 10b. The vacancies deplete with a slope in the log *c* plot that is twice as steep as for the holes as the vacancies are doubly charged compared with the single charged holes. The concentration of the dopant *c*_dop_ (in nominally undoped STO, cation vacancies) is assumed to be constant due to its low mobility in the temperature range considered here. For a single depleted defect, this is commonly known as the Mott–Schottky model, and this is also the basis behind eqs 1 and 2 and thus the calculated Δϕ_EIS_ and *w*_SC_ values.

### Dependence of Δϕ on the Specific MIEC

A more detailed look at eq 18 gives a better understanding of how and why Δϕ changes in dependence of the chosen MIEC atop of the STO. For the sake of simplicity, we consider a case with site restriction for holes and vacancies only in the MIEC and no further defect interactions. We thus get for the bulk defect concentrations
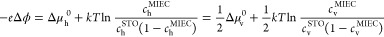
19As mentioned above, we can describe the expected space charge potential equivalently either from the viewpoint of electronic or ionic defects, and in either case, the space charge potential is determined by a standard chemical potential term and a concentration term. In the following, we apply this model to our measured space charge potentials and explain the observed trends in materials dependence semiquantitatively. First, we exemplify the two alternative views on Δϕ, either via holes or via vacancies.

For all space charges measured in this study, nominally identical STO single crystals were used, with one and the same μ_h_^0,STO^ and  for given *p*(O_2_). The absolute hole concentration [h^·^], for example, is approximately 7 × 10^15^ cm^3^ at 500 °C and 1 bar O_2_ and a doping level of ca. 24 ppm.^26^ Since all solid solutions for the used MIECs can be considered highly doped materials (doping concentration between 0.1 and 0.4 per unit cell, i.e., 4 orders of magnitude higher than STO) and all are hole conductors at high oxygen partial pressures, the logarithmic term considering the hole concentration should be very similar for all measured perovskite-type electrode materials. Accordingly, the main reason behind the variations of Δϕ for the different MIECs is their different , i.e., different valence band edges , or, in the case of polaronic conduction, different polaron energy levels. Hence, our variation of Δϕ reflects the differences of valence band edges or polaron levels in the MIECs. (Please note that for metallic MIECs such a simple split into concentration and standard terms fails.) The remarkably high space charge potentials in the 1 V range for LSM and LSCr are thus also largely due to the much lower valence band edge in STO. According to eq 19, our model predicts an even higher Δϕ for acceptor-doped STO, due to an increase in .

From the viewpoint of oxygen vacancies, we first assume that  is similar for all investigated perovskite-type MIECs and thus has little influence on the variation of Δϕ in dependence of the MIEC on top of STO. At ambient pressure, vacancies are the minority charge carriers in the investigated MIECs, and their concentrations may differ by several orders of magnitude. Since  is the same in all experiments (at given O_2_),  is the determining factor in Δμ_v_ and therefore in Δϕ. Hence, the different space charge potentials also reflect the very different oxygen vacancy concentrations in, for example, LSF and LSM.

In order to better understand the decisive differences of  in the electronic picture or  in the ionic view, a detailed look at the defect chemical models of the oxygen exchange reaction is helpful. For the highly doped MIEC, one can write the equilibrium of the oxygen exchange reaction as follows:
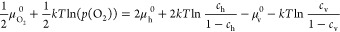
20Rearrangement of eq 20 leads to
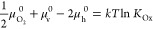
21with the mass action constant of the oxygen exchange reaction (eq 10) defined as
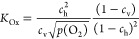
22For the dilute situation of STO the (1 – *c*) terms in eqs 20 and 22 can be neglected. Combining eqs 21 and 16 results in
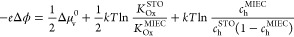
23(Please note that the standard chemical potential  for gaseous O_2_ is the same in all experiments and thus  is zero.) Owing to the very different dopant levels, we can expect a significant difference of  between STO and the MIECs and thus substantial  values. However, the similarly doped MIECs might also have similar  values due to similar interaction of oxygen vacancies, polaronic holes, and Sr doping, even though experimental or theoretical evidence of this hypothesis is still missing. Assuming its validity, we can expect similar  values for all our MIECs. Since the doping concentration *c*_dop_ and thus  is also similar in our study, this suggests a direct relationship between the space charge potential and the reducibility of the MIECs, given by : The better reducible, the smaller  and the smaller the positive Δϕ.

If, for example, μ_h_^0^ is lowered and thus the polaronic level in Figure 9 is increased, *K*_Ox_ in eq 21 increases, and it is more favorable for the MIEC to form holes compared to an MIEC with a higher μ_h_^0^. An easier formation of holes for an MIEC means more pronounced oxidation of the metallic cations or less reducibility. In defect chemical models, i.e., Brouwer diagrams, a low  of acceptor-doped MIECs thus translates to a dominant concentration of holes down to low *p*(O_2_). This reducibility of MIECs is also highly important when these materials are applied in solid-oxide fuels or electrolysis cells. It determines the range of high- or low-oxide ion and hole conductivities.

We may now define partial pressure *p*(O_2_)* where the majority charge carrier counterbalancing the singly charged absolute dopant concentration [dop] changes from holes to oxygen vacancies, i.e., for absolute concentrations, we find . In terms of normalized concentrations, this corresponds to , and from eq 22, we get
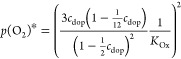
24(Please note that the vacancy concentration *c*_v_ is normalized to three sites per perovskite unit cell, while *c*_h_ is normalized to one site per unit cell for polaronic MIECs. In the case of band conduction, the prefactors of 3 and  in eq 24 change in accordance with the density of states at the valence band edge.) This pressure *p*(O_2_)* is thus a good measure of the reducibility of an MIEC. Combining eqs 21 and 24 leads to
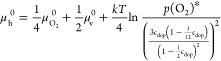
25A low *p*(O_2_)* indicates a low  and, in turn, a high *p*(O_2_)* corresponds to a high . STO Brouwer diagrams reveal a *p*(O_2_)* far above 1 bar,^26^ suggesting a high  and thus a low valence band edge. Calculations and measurements of the LSF Brouwer diagram^27^ reveal primarily holes at ambient conditions, and thus  of LSF is likely lower than that of STO. (Here, we neglect additional effects due to different , e.g., caused by the very different dopant levels.) Moreover, it is well-known in the solid-oxide fuel cell community that *p*(O_2_)* for LSM and LSCr is far lower than that for LSF as LSCr and LSM have to be exposed to very reducing conditions in order to release oxygen, thereby annihilating holes. Therefore,  of LSCr and LSM is also much lower.

**Figure 11:**
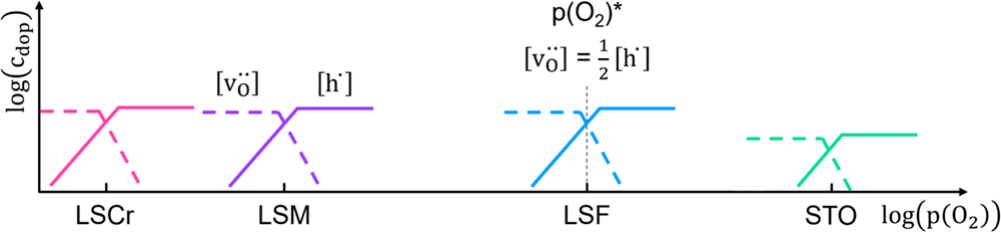
Sketched series of *p*(O_2_)* illustrated by simplified Brouwer diagrams of the acceptor-doped perovskite-type oxides LSCr, LSM, LSF, and STO. Defect interaction is neglected, and only oxygen vacancies  and holes [h^·^] are shown. Distances between the different *p*(O_2_)* points are not drawn to scale.

The decisive parts of the corresponding Brouwer diagrams of our acceptor-doped perovskite-type oxides are sketched in Figure 11; defect interaction is neglected. According to eq 25 and the suggested series of *p*(O_2_)* values,  should thus display the following trend (eq 26)
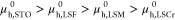
26and the opposite series holds for the valence band edge or the hole polaron level. With our assumption that  determines the difference in μ_h_ of the MIECs and Δμ_h_ = −*e*Δϕ, the space charge potential Δϕ between an MIEC and STO should thus follow the trend (eq 27)
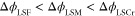
27This is in accordance with the experiments. LSC behaves similar to LSF due to its similar reducibility (see below), even though its defect chemistry is determined by its metallic character.

The suggested qualitative correlation of space charge potential and reducibility can also be tested by using thermodynamic data found in the literature. The equilibrium constant *K*_Ox_ is given by eq 28
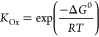
28where R is the universal gas constant, and the Gibbs free energy for oxygen incorporation Δ*G*^0^ is given by eq 29
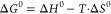
29Using thermodynamic data from literature, i.e., reaction enthalpy Δ*H*^0^ and entropy Δ*S*^0^ values of reaction (eq 10) for LSF,^53^ LSM,^54^ and LSCr,^55^*K*_Ox_ can be calculated for 500 °C, i.e., the temperature used during impedance measurements. Please note that a range of thermodynamic data is reported in the literature. We chose the sources that match best the results for our thin films. Deviations to true *K*_Ox_ values at 500 °C may occur, as the given experimental Δ*H*^0^ and Δ*S*^0^ values were partly determined at temperatures around 1000 °C. Moreover, defect thermodynamic data of thin films may differ from those of bulk material.^68^ The values used here as well as calculated *p*(O_2_)* values (eq 24) are summarized in Table 3.

**Table 3:** Thermodynamic Data from the Literature Used to Calculate the Given *p*(O_2_)* from Eq 24 Where Hole Compensation of Acceptor Dopant Switches to Oxygen Vacancy Compensation^a^

	LSF	LSM	LSCr
Δ*H*^0^ (kJ mol^–1^)	–95.62 ± 4.18	–271.46 ± 5.871	–303.1
Δ*S*^0^ (J mol^–1^K^–1^)	–54.27 ± 4.43	–135 ± 5	–100.5
reference	Kuhn^53^	Tanasescu^54^	Mizusaki^55^
*c*_dop_	0.4	0.35	0.1
*p*(O_2_)* (bar)	1.8 × 10^–7^	5.9 × 10^–23^	3.8 × 10^–32^

a The dopant concentration *c*_dop_ for the materials is given as well.

**Figure 12:**
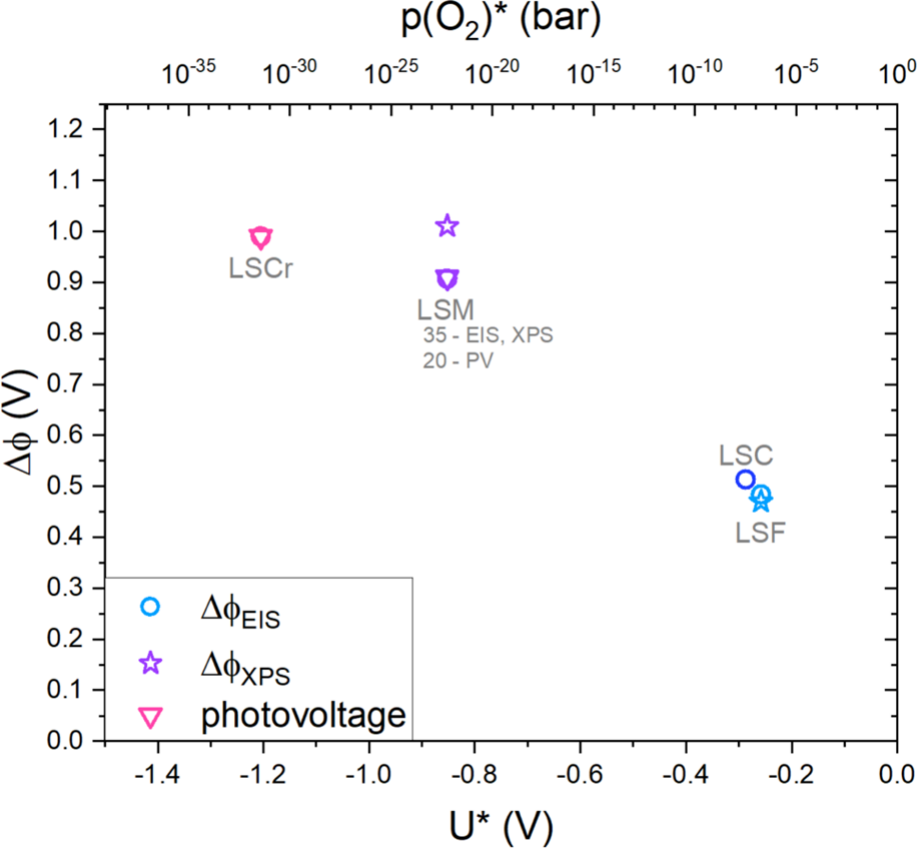
Plot of the space charge potential calculated from impedance data Δϕ_EIS_ at 2 mbar and XPS data Δϕ_XPS_ at 1 mbar against the reducibility U* at 500 °C.

By means of Nernst’s eq 30, we can express the reducibility also in terms of voltage *U* with respect to 1 bar O_2_ according to
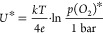
30The calculated reduction voltages U* amount to −0.258 V for LSF, −0.852 V for LSM, and −1.205 V for LSCr, which is equivalent to a *p*(O_2_)* of 1.8 × 10^–7^ bar, 5.9 × 10^–23^ bar, and 3.8 × 10^–32^ bar, respectively. In Figure 12, these reducibilities are compared to space charge potentials Δϕ_EIS_ and Δϕ_XPS_, which have been measured at an oxygen partial pressure of 2 × 10^–3^ and 1 × 10^–3^ bar, respectively. The errors of Δ*H*^0^ given in the literature (Table 3) transfer to errors of U* in the 10 mV range. The lower *x*-axis shows the calculated reduction voltage U*, while the upper *x*-axis displays the corresponding *p*(O_2_)* (bar). The absolute value of U* is the lowest for LSF followed by LSM, and U* is the highest for LSCr. Notably, the reducibility indeed follows the same trend as the space charge potential: LSF < LSM < LSCr, meaning that the reducibility can be used as a helpful tool to predict trends of space charges between MIECs. Actually, also LSC fits well to this scheme. It releases substantial oxygen at similar *p*(O_2_) values as LSF^53,69^ and thus exhibits a similar reducibility, even though simple defect chemical models cannot be applied. We may still define a *p*(O_2_)* value by the condition , and from ref (69), a value of ca. 3.15· 10^–8^ bar can be approximated at 500 °C for 30% Sr doping. This value is also plotted in Figure 12. Accordingly, the LSF and LSC exhibit similar space charge potentials. However, also considering YBCO by means of a similar approach is beyond the scope of this paper.

The applicability of our model becomes even more obvious when expressing the difference of two space charge potentials ΔΔϕ = Δϕ^MIEC II^ – Δϕ^MIECI^ in terms of U*. According to eq 23, we have eq 31
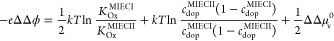
31Together with eq 24 we get eq 32
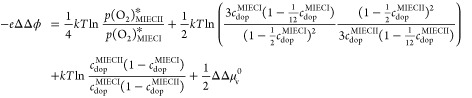
32and thus eq 33
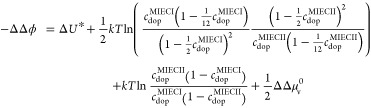
33For the same doping concentration this simplifies to eq 34
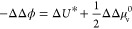
34

From Figure 12, we see that ΔU* between LSM and LSF is 0.59 V and thus rather close to the experimental ΔΔϕ value, which is 0.43 V for impedance data and 0.54 V from XPS. In principle, the difference between ΔΔϕ and ΔU* could even be used to determine , even though the accuracy of Δϕ and ΔU* is probably not sufficient to give a reliable value for our specific data. The difference of ΔU* between LSM and LSCr is larger than expected from measured Δϕ values, which, however, may be due to several reasons, including shortcomings of the high-temperature thermodynamic data, differences in , errors due to the simplified defect model, or also due to other assumptions such as absence of interfacial charges and dipoles. However, we are optimiztic that future accurate measurements of *K*_Ox_ under the very same experimental conditions of the Δϕ measurements will further illuminate the close relationship between space charges and reducibilities and may even lead to experimental  data. Moreover, these approaches to relate reducibilities to space charge potentials are not limited to the materials used here but are expected to also hold for many other combinations of mixed-conducting oxides and even for predominantly ionic-conducting oxides. Therefore, considering vacancies instead of holes is presumably advantageous for predictions of Δϕ.

## Conclusions

LSF, LSC, LSM, LSCr, and YBCO thin films were deposited on STO (100) single crystals using PLD, and space charge potentials of the resulting MIEC|STO interfaces were characterized. In impedance spectroscopic measurements, a moderate space charge potential of about 0.5 V was found for LSF and LSC, while LSM and LSCr showed very pronounced space charge potentials in the 1.0 V range. YBCO does not show a measurable space charge on STO. The space charge potentials Δϕ_EIS_ thus follow the trend LSF ≤ LSC < LSM < LSCr. Space charge potentials of LSF|STO and LSM|STO interfaces were also characterized by NAP-XPS measurements. Here, a method was developed allowing quantitative space charge potentials to be obtained by comparing binding energies with those measured for Nb:STO. These XPS measurements clearly confirmed the trend found for Δϕ_EIS_. Further comparison with literature data on photovoltages measured for nominally identical LSM|STO and LSCr|STO junctions support the validitiy of the space charge potential values.

A model is proposed to describe the combined response of ionic and electronic defects at the interface between two mixed ionic and electronic conductors. The model is based on the assumption of an equilibrium of both MIECs with the surrounding oxygen partial pressure. For two different acceptor-doped MIECs, this leads to a different chemical potential of both holes and oxygen vacancies in the two phases and thus to a space charge region to equilibrate this difference in the respective defect chemical equilibrium. Interestingly, defect thermodynamic considerations imply that an equilibrium space charge may be completely characterized by only considering holes or by only considering vacancies since  holds in equilibrium with the gas phase. This also means that ions and electrons mutually form the same space charge. For similar hole concentrations in the MIECs, different space charge potentials of the highly doped MIECs on STO largely reflect differences in the valence band edges or polaron levels. More detailed defect chemical considerations reveal that there is a strong relation between the space charge and the reducibility of a mixed conductor, i.e., the oxygen partial pressure *p*(O_2_)* at which the hole compensation of the dopant changes to vacancy compensation. It is demonstrated that larger space charge potentials at the MIEC|STO interface are expected for less reducible oxides, such as LSM and LSCr, which is in excellent agreement with the presented experiments. A quantitative model was developed to predict differences in space charge potentials from thermodynamic reduction potentials, dopant levels, and differences in the standard chemical potential of oxygen vacancies . The model demonstrates that Brouwer diagrams of MIECs as well as space charge potentials are closely related since both are based on the same defect thermodynamic data.
